# AAPM Medical Physics Practice Guideline 14.a: Yttrium‐90 microsphere radioembolization

**DOI:** 10.1002/acm2.14157

**Published:** 2023-10-11

**Authors:** Nathan C. Busse, Muthana S. A. L. Al‐Ghazi, Nadine Abi‐Jaoudeh, Diane Alvarez, Ahmet S. Ayan, Erli Chen, Michael D. Chuong, William A. Dezarn, Shirin A. Enger, Stephen A. Graves, Robert F. Hobbs, Mary Ellen Jafari, S. Peter Kim, Nichole M. Maughan, Andrew M. Polemi, Jennifer R. Stickel

**Affiliations:** ^1^ Colorado Associates in Medical Physics Denver Colorado USA; ^2^ University of California Irvine California USA; ^3^ Department of Radiological Sciences University of California Irvine California USA; ^4^ Baptist Hospital Miami Cancer Institute Miami Florida USA; ^5^ Department of Radiation Oncology Ohio State University Columbus Ohio USA; ^6^ Department of Radiation Oncology Cheshire Medical Center Keene New Hampshire USA; ^7^ Department of Radiation Oncology Miami Cancer Institute Miami Florida USA; ^8^ Department of Radiation Oncology Wake Forest University School of Medicine Winston‐Salem North Carolina USA; ^9^ Department of Oncology McGill University Montreal Canada; ^10^ Department of Radiology University of Iowa Iowa City Iowa USA; ^11^ Department of Radiation Oncology Johns Hopkins University Baltimore Maryland USA; ^12^ Diagnostic Physics, Atlantic Health System Morristown Medical Center Morristown New Jersey USA; ^13^ Medical Physics Unit McGill University Montreal Canada; ^14^ Department of Radiation Oncology Washington University in St. Louis Saint Louis Missouri USA; ^15^ Department of Radiology University of Virginia Charlottesville Virginia USA

**Keywords:** brachytherapy, hepatic tumors, MPPG, practice guideline, radioembolization, Yttrium‐90

## Abstract

Radioembolization using Yttrium‐90 (^90^Y) microspheres is widely used to treat primary and metastatic liver tumors. The present work provides minimum practice guidelines for establishing and supporting such a program. Medical physicists play a key role in patient and staff safety during these procedures. Products currently available are identified and their properties and suppliers summarized. Appropriateness for use is the domain of the treating physician. Patient work up starts with pre‐treatment imaging. First, a mapping study using Technetium‐99^m^ (Tc‐99^m^) is carried out to quantify the lung shunt fraction (LSF) and to characterize the vascular supply of the liver. An MRI, CT, or a PET‐CT scan is used to obtain information on the tumor burden. The tumor volume, LSF, tumor histology, and other pertinent patient characteristics are used to decide the type and quantity of ^90^Y to be ordered. On the day of treatment, the appropriate dose is assayed using a dose calibrator with a calibration traceable to a national standard. In the treatment suite, the care team led by an interventional radiologist delivers the dose using real‐time image guidance. The treatment suite is posted as a radioactive area during the procedure and staff wear radiation dosimeters. The treatment room, patient, and staff are surveyed post‐procedure. The dose delivered to the patient is determined from the ratio of pre‐treatment and residual waste exposure rate measurements. Establishing such a treatment modality is a major undertaking requiring an institutional radioactive materials license amendment complying with appropriate federal and state radiation regulations and appropriate staff training commensurate with their respective role and function in the planning and delivery of the procedure. Training, documentation, and areas for potential failure modes are identified and guidance is provided to ameliorate them.

## 1 DECLARATION

The American Association of Physicists in Medicine (AAPM) is a nonprofit professional society whose primary purposes are to advance the science, education, and professional practice of medical physics. The AAPM has more than 8,000 members and is the principal organization of medical physicists in the United States.

The AAPM will periodically define new practice guidelines for medical physics practice to help advance the science of medical physics and to improve the quality of service to patients throughout the United States. Existing medical physics practice guidelines will be reviewed for the purpose of revision or renewal, as appropriate, on their fifth anniversary or sooner.

Each medical physics practice guideline represents a policy statement by the AAPM, has undergone a thorough consensus process in which it has been subjected to extensive review, and requires the approval of the Professional Council. The medical physics practice guidelines recognize that the safe and effective use of diagnostic and therapeutic radiology requires specific training, skills, and techniques, as described in each document. Reproduction or modification of the published practice guidelines and technical standards by those entities not providing these services is not authorized.

The following terms are used in the AAPM practice guidelines:

- Must and Must Not: Used to indicate that adherence to the recommendation is considered necessary to conform to this practice guideline. While must is the term to be used in the guidelines, if an entity that adopts the guideline has shall as the preferred term, the AAPM considers that must and shall have the same meaning.
- Should and Should Not: Used to indicate a prudent practice to which exceptions may occasionally be made in appropriate circumstances.

## 2 DEFINITIONS

Definitions, terms, and acronyms used in this document are listed below:

**Table:** 

ALARA	As Low As Reasonably Achievable, social and economic factors permitting
AU	Authorized user who is listed on the institution's radioactive materials license and meets the requirements stipulated by the Nuclear Regulatory Commission (NRC) or Agreement States. This is often a board‐certified radiation oncologist, a board‐certified interventional radiologist, or a board‐certified nuclear medicine physician.
Bq	Becquerel
BSA	Body surface area
CBCT	Cone Beam Computed Tomography ‐ an imaging technique employing divergent x‐rays with a flat‐panel detector for three‐dimensional reconstruction akin to computed tomography (CT). Whenever specified in this report, multidetector CT would also suffice.
CFR	Code of Federal Regulations
Child‐Pugh score	The Child‐Pugh score is a system for assessing the prognosis — including the required strength of treatment and necessity of liver transplant — of chronic liver disease, primarily cirrhosis.
CHP	Certified Health Physicist as recognized by the American Board of Health Physics
Ci	Curie
DVH	Dose‐volume histogram
FMEA	Failure mode and effect analysis
HCC	Hepatocellular carcinoma
IR	Interventional radiology
LSF	Lung shunt fraction
MAA	Macroaggregated albumin
MPPG	Medical Physics Practice Guideline
NCCN	National Comprehensive Cancer Network
NRC	Nuclear Regulatory Commission
PET	Positron Emission Tomography
QMP	Qualified medical physicist properly trained to support Yttrium‐90 (^90^Y) treatment as defined in AAPM position statement PS 7‐A. For 90Y treatments, certification in any of the specialties listed in PS 7‐A are appropriate to serve as a QMP. While QMPs are required for dosimetry calculations, day of treatment radiation safety support may be provided by appropriately trained certified health physicists or radiation safety staff.
RAM	Radioactive Materials
REILD	Radioembolization‐induced liver disease
ROI	Region of Interest
S value	Mean absorbed dose per cumulated activity. S values depend on the radionuclide, source organ, target organ, and geometry inherent from the selection of the age and gender of phantom selected.
SIRT	Selective internal radiation therapy
SPECT	Single photon emission computed tomography
TARE	Transarterial radioembolization
TNR [T/N]	Tumor to normal tissue ratio of the liver
WD	Written Directive

## 3 INTRODUCTION

### 3.1 Introduction

Radioembolization, also referred to as transarterial radioembolization (TARE) or selective internal radiation therapy (SIRT), is a form of brachytherapy for primary and secondary cancers in the liver. It is predominantly used for the management of multifocal and/or very large intrahepatic tumor burden not feasibly treated with surgery or other liver‐directed therapies. Yttrium‐90 (^90^Y) is a pure beta emitting radioisotope with a 64.04 hour half‐life that can be attached to glass or resin microspheres in a colloidal suspension. Radioembolization treatment involves multiple clinical steps connecting multidisciplinary fields. In ^90^Y TARE, microspheres are injected into the hepatic arterial supply through a microcatheter. Spheres become lodged in the hepatic vascular bed and irradiate tumorous tissue with energetic beta particles. This document develops a set of guidelines to ensure safe and effective utilization of this technology. This is the first attempt at establishing guidelines for ^90^Y microsphere therapy since the publication of TG‐144 in 2011.^1^ These guidelines provide the necessary information on:

1. institutional radioactive materials (RAM) licensing and regulatory compliance
2. authorized user (AU) and qualified medical physicist (QMP) qualifications
3. staff training and safety guidelines
4. pre‐treatment patient evaluation and imaging studies
5. preparation and administration of ^90^Y microspheres
6. post‐treatment imaging, treatment location verification, and absorbed dose evaluation
7. patient safety and discharge instructions
8. radioactive waste disposal

### 3.2 Clinical indications and patient selection

TARE achieves excellent therapeutic ratio by selectively delivering microscopic radioactive particles into the terminal arterioles of liver tumors. This natural bias towards tumor irrigation is a product of the dual blood supply to the liver: normal tissue tends to receive more blood supply from the portal vein, while the tumor preferentially draws from the hepatic artery. The therapeutic ratio effectively improves intrahepatic tumor control while minimizing the relative risk of radioembolization‐induced liver disease (REILD) even for patients with extensive tumor burden.^2^, ^3^ The possibility of using combined therapies has also been recommended in the literature.^4^, ^5^

There is a large body of prospective and retrospective literature demonstrating both safety and efficacy of radioembolization for the management of primary^2^, ^6^, ^7^, ^8^ and metastatic^3^, ^9^, ^10^ liver cancers. The literature for colorectal liver metastases, especially patients with liver only, or liver dominant disease, is particularly extensive. It demonstrates both efficacy and safety for patients treated predominantly in the salvage setting after progression on systemic therapy.^3^, ^9^  In the first line setting, randomization data demonstrated significantly improved intrahepatic tumor control compared to chemotherapy alone, without a benefit in overall survival or progression free survival. There was, however, a potential overall survival benefit for patients with right‐sided colon cancers.^10^  Certain patients with non‐colorectal liver metastases may benefit significantly from radioembolization. This is based on mostly single center retrospective data.^7^  TARE has been extensively studied over many decades for carefully selected patients with unresectable hepatocellular carcinoma (HCC).^11^ It is endorsed by the National Comprehensive Cancer Network (NCCN) guidelines for the management of unresectable HCC and metastases originating from colorectal cancer and neuroendocrine tumors.^12^

While patient selection is outside the scope of a physicist's practice, it is critical to minimize the risk of potentially severe or fatal toxicity.^13^ Some background is provided here. Patients’ bilirubin should be < 2 mg/dL and ideally below 1.3 mg/dL if lobar or bi‐lobar treatment is planned.^14^, ^15^, ^16^ Most publications indicate that greater latitude with higher levels of bilirubin may be acceptable when selective radiation segmentectomy is performed.^15^, ^17^ Albumin levels and trends are also important since they tend to decrease before changes in bilirubin appear. Decreasing albumin levels forecast impending liver dysfunction. Albumin levels > 3 g/dL are associated with improved survival post‐TARE.^6^, ^15^, ^18^

A key consideration is to treat with caution patients with significantly decompensated liver function (e.g., Child‐Pugh score ≥ B8). Other relative contraindications include greater than 70%–75% liver involvement by tumor, poor performance status, pregnancy, or a high lung shunt fraction (LSF).

Presence of ascites, especially uncontrolled, is an indicator of poor outcome. Infiltrative disease with a tumor burden greater > 50%, ECOG (Eastern Cooperative Oncology Group) performance status > 2, and, in patients with cirrhosis, a liver volume < 1.5L have been associated with liver failure post‐TARE and are considered relative contra‐indications to the treatment.^6^, ^9^, ^16^ Although no definite prospective data exist on the matter, several large studies demonstrated a correlation between increased lines of prior chemotherapy and REILD.^9^, ^17^

Prior radiation therapy to the liver requires careful planning to ensure patient safety. Radioembolization may be reasonable especially if given in a segmental fashion after careful assessment of the prior absorbed dose delivered to the liver.^19^   Prior therapies can affect residual liver function and predispose to REILD.

Contrast enhanced liver protocol CT or MRI should be used for lesion disease burden determination and identification of imaging factors associated with poor prognosis such as ascites.

### 3.3 Currently available products

At the time of this writing there are two commercially available ^90^Y microsphere products; TheraSphere™ Y‐90 glass microspheres; and SIR ‐Spheres® resin microspheres. Pertinent characteristics of these products are summarized in Table 1.

**TABLE 1:** Characteristics of therasphere and SIR‐spheres.

Parameter	Glass (TheraSphere)	Resin (SIR‐Spheres)
Manufacturer	Boston Scientific, Marlborough, MA	Sirtex Medical Inc., Wilmington, MA
Size	20–30 μm	22–42 μm
Isotope	^90^Y integrated within glass matrix	^90^Y on resin surface
Specific gravity	High	Low
Activity per sphere at calibration	2500 Bq	50 Bq
Activity per sphere at treatment	100–1500 Bq	52–148 Bq
Available activity vials at date and time of calibration	3–20 GBq vials	3 GBq vials
Time of calibration	Prior to treatment	Day of treatment or up to three days after
# spheres/vial	1.2–8 Million	44 ± 2.6 Million
# spheres/3GBq activity	1.2 Million	44 ± 2.6 Million (3 GBq vial)
US—FDA Approval [year]	Hepatocellular carcinoma [2021]	Colorectal metastases [2003]

### 3.4 Information for administrators

Administrators over the relevant departments including interventional radiology (IR), nuclear medicine, and radiation oncology should review Section 1 to obtain a basic knowledge of ^90^Y microsphere clinical applications and workflow. ^90^Y microsphere therapy is a complex procedure that requires a coordinated multidisciplinary team. This team typically involves a combination of an interventional radiologist, nuclear medicine physician, radiation oncologist, QMP, nuclear medicine technologist (NMT) and a host of other support staff. All staff must have adequate training for the procedure. The practice should aim to maintain a minimum of three cases per year before starting a ^90^Y program. All relevant policies and procedures must be in place before performing a clinical case.

Administrators must obtain the services of a QMP. This individual must have specific training regarding ^90^Y radioembolization and must meet the qualifications defined by their RAM license to support ^90^Y treatment. The QMP is a key member of the ^90^Y care team and is responsible for the correct determination of the ^90^Y activity required, its safe preparation and delivery, safety of the patient and staff, and familiarity and compliance with applicable state and federal regulations. The QMP's role includes assessing the quantitative accuracy of the administered activity and absorbed dose delivered to the patient, particularly where image‐based dosimetry is used. Some states mandate the presence of a QMP for ^90^Y procedures. All dosimetry requires the services of a QMP. Appropriately trained certified health physicists (CHP), RSOs, or other radiation staff members may support day‐of‐treatment activities such as dose preparation and post‐treatment surveys. Any radiation safety tasks that may be supported by non‐QMPs will be stated explicitly. See Section 3.E for details on QMP and RSO training.

The QMP must work closely with the treating physician AU, radiation safety personnel, nuclear medicine staff, IR staff, nursing personnel, other team members, and the vendor(s) to ascertain that a safe and efficacious program is in place and compliant with applicable radiation regulations. The QMP and/or RSO must evaluate staff competency regarding radiation safety and other technical aspects of the procedure and/or provide retraining every three months if the program treats fewer than one ^90^Y patient per quarter.

Several diagnostic studies must be performed prior to deciding to proceed with ^90^Y microsphere therapy. These generally include contrast‐enhanced CT or magnetic resonance imaging (MRI), digital subtraction angiography (DSA), Cone Beam CT (CBCT), ^99m^Tc‐MAA gamma camera planar imaging and single photon emission computed tomography (SPECT). Billing for these scans should be done in accordance with accepted criteria within IR and nuclear medicine departments. A trained medical biller ensures that the ^90^Y microsphere therapy technical billing for the service is appropriately completed. Relevant billing codes for a ^90^Y microsphere therapy procedure at the time of writing are tabulated in Table 2. In addition, the American Society for Radiation Oncology (ASTRO) annually publishes a Radiation Oncology Coding Resource.

**TABLE 2:** Yttrium‐90 procedure codes, description and usage guidelines.

Procedure code	Description	^90^Y specific guidance
77263	Complex therapeutic radiology treatment planning	Can be reported by IR Radiologist or Radiation Oncologist but not both
77290	Complex therapeutic radiology simulation	Placement of a catheter into intrahepatic vascular system
77295	3‐dimensional isodose plan	Must meet the medical necessity requirement and plan detail must rise to the level of a 3‐D plan with defined CTVs, OARs and able to report DVH
77300	Basic dosimetry calculation	Calculation by a medical physicist for the activity to be administered, MIRD uniform or partition model dose‐based planning.
77318	Complex brachytherapy isodose plan	Image based isodose plan, includes 77300
77370	Special medical physics consultation	Requested by physician, performed by QMP, approved by physician and report placed in medical record
77470	Treatment management for radiation procedures requiring extensive planning	Reported by IR Radiologist or Radiation Oncologist but not both
77778	Complex interstitial source application, includes supervision, handling, loading of radiation source	Once per session, regardless of the number of infusions performed, reported by radiation oncologist
77790	Supervision, handling, loading of radiation source	Do not report with 77778
78201	A liver and spleen scan is a specialized radiologic procedure that is used to examine the liver and spleen to identify certain conditions or to assess their function.	
78580	Lung perfusion imaging	
78800	Planar lung shunt assessment, single area, single day imaging	Do not report with 78801 or 78804
78801	Planar lung shunt assessment, two or more areas	Do not report with 78800 or 78804
78803	SPECT, single area, single day imaging	Do not report with 78830
78804	Planar, whole body, requiring two or more days imaging	Do not report with 78814
78814	Limited FoV PET/CT lung shunt and liver function post treatment assessment	Do not report with 78804
78830	SPECT with concurrently acquired CT, single area, single day imaging	Do not report with 78803, 78831, or 78832
78831	SPECT only, single area over two or more days or minimum two areas in single day imaging	Do not report with 78803, 78830, or 78832
78832	SPECT with concurrently acquired CT, single area over two or more days or minimum two areas in single day imaging	Do not report with 78803, 78830, or 78831
78835	Radiopharmaceutical quantification measurement(s) single area, report multiple units for more than one area or more than one day of imaging	Use with 78830 or 78832
79445	Radiopharmaceutical therapy intra‐arterial particulate delivered by IR	Reported by IR AU and do not report with 77778
99211‐99215	Follow up evaluation and management	
99223	Initial consultation	Determine treatment eligibility
A9540	HCPCS code for Tc‐99 m	Pass through cost, per study dosage (activity), up to 10 mCi
C2616	Brachytherapy source, non‐stranded, ^90^Y	Pass through cost
S2095	Transcatheter occlusion or embolization for tumor destruction, percutaneous, any method, using ^90^Y	Some carriers require use of this procedure code instead of CPT code 77778

**FIGURE 1:**
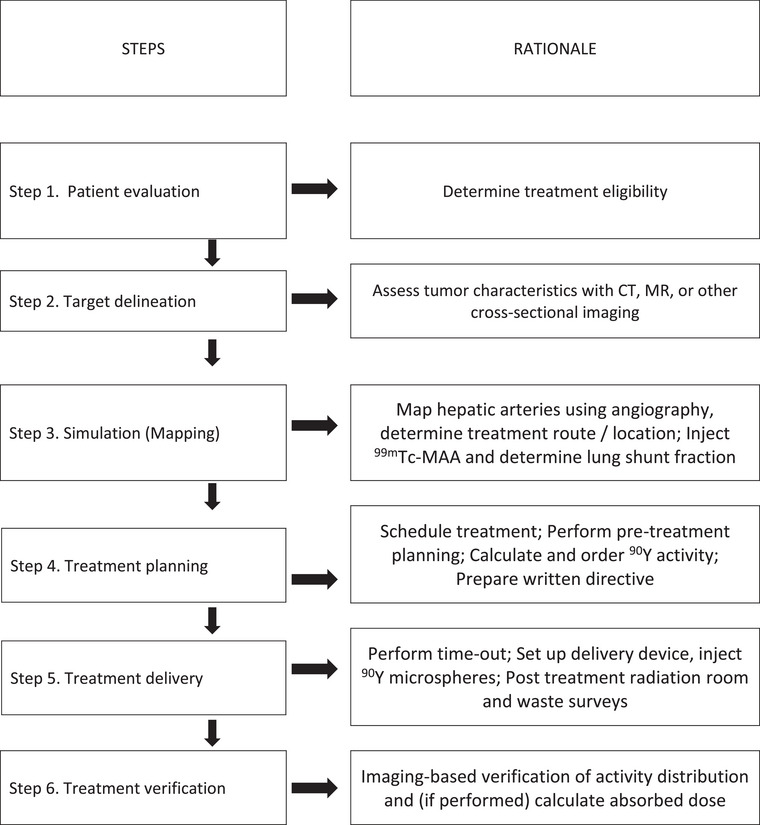
Typical ^90^Y radioembolization procedure workflow.

Figure 1 illustrates the workflow of a ^90^Y procedure. An alternate workflow is available in Kim et al.^20^

## 4 REGULATORY COMPLIANCE

### 4.1 Radioactive materials licensing

#### 4.1.1 License amendment

A RAM license amendment is required for specific licensees prior to clinical ^90^Y radioembolization treatment. The U.S. Nuclear Regulatory Commission (NRC) and Agreement States regulate and license ^90^Y microsphere therapy under 10 CFR 35.1000, “Other medical uses of byproduct material or radiation from byproduct material” (U.S. NRC, 2020).^21^

A guidance document, Yttrium‐90 Microsphere Brachytherapy Sources and Devices TheraSphere and SIR‐Spheres Licensing Guidance, was initially published by the NRC in October 2002, and most recently revised in 2019 (revision 10), with a short additional revision (10.2) issued April 20, 2021. The applicant should follow instructions in the most recent revision of the licensing guidance document, available on the NRC medical toolkit website, to request a radioactive materials license amendment. Not all Agreement States follow the NRC licensing guidance, so licensees should reach out to their licensing authority if they are located in an Agreement State to ensure they are using the applicable licensing guidance for their state. The amendment request must include the radionuclide, chemical/physical form, requested maximum possession limit, purpose of use, facility address and description, names of the requested AUs and documentation of their training and experience applicable to ^90^Y radioembolization, and the name of the Radiation Safety Officer (RSO) and documentation of his/her training in radiation safety, regulatory issues, and emergency procedures for ^90^Y microsphere use.

The license amendment request must include commitments to follow all the requirements in 10 CFR Part 35 for brachytherapy sources and manual brachytherapy use as applicable, and a commitment to follow procedures for administration specific to ^90^Y radioembolization as given in the current guidance document.

If a new revision of the guidance document is issued by the NRC, licensees committed to a previous version must request a license amendment to follow the new revision if they are not authorized by their license to make radiation protection program changes to a new guidance. This license amendment must be applied for and received before the licensee can make radiation protection program changes to conform to the new guidance. The applicant or licensee may request to incorporate a change process into its license, which permits future changes to radiation safety programs without a license amendment. The procedure for requesting this change process is detailed in the NRC guidance document.

#### 4.1.2 AU qualifications

AUs must meet the training and experience requirements in the most recent version of the NRC licensing document or applicable Agreement State guidance. Licensees may also submit alternative training and experience for approval on a case‐by‐case basis by NRC or Agreement State staff. The alternative training and experience request for approval should include an explanation of why the applicant believes the alternative qualifications demonstrate that the individual is qualified to be an AU.

The NRC recognizes that, if an AU satisfies the training and experience requirements listed in NRC's licensing guidance for ^90^Y microspheres and is currently listed on an NRC or Agreement State medical use license or permit for a specific type of microsphere, the AU should be allowed to work under a different license for the medical use of the same type of microsphere. A limited specific medical use applicant may request authorization to submit notification of the new AU to the NRC instead of an amendment request, provided conditions detailed for this situation in the NRC licensing document are met.

#### 4.1.3 Radiation safety officer qualifications

The RSO must have training as specified in 10 CFR 35.50, including training in radiation safety, regulatory issues, and emergency procedures for ^90^Y microsphere use. A RSO already listed on a license that includes one type of ^90^Y microsphere device does not require additional approval for another type of ^90^Y microsphere device, but they should be familiar with all radiation safety aspects for all microsphere products used at the facility.

### 4.2 Waste disposal

Yttrium‐90 microspheres may contain radioactive impurities, some of which have half‐lives longer than 120 days.^22^ Impurities are not required to be listed on an NRC license, but applicants are responsible to ensure the microspheres are handled and disposed of in accordance with 10 CFR Part 20 and Part 35 requirements. Specifically, 10 CFR 35.92 requires that licensees monitor byproduct material with a physical half‐life of less than or equal to 120 days. Before disposal, the activity must be low enough that it cannot be distinguished from background, when measured with an appropriate, calibrated radiation detection survey meter. Licensees may need to hold the waste for an extended time or transfer the ^90^Y microspheres to an authorized recipient pursuant to requirements in 10 CFR Parts 20 and 30.

### 4.3 Instrumentation and surveys

Licensees are required to measure and record the activity of each dosage before medical use. An appropriate dose calibrator must be used with a dose calibrator setting specific to the product for measurement of ^90^Y activity (see Section 6). Licensees must have appropriate, calibrated radiation detection and measurement equipment. A Geiger‐Mueller survey meter is sufficient for surveys to determine the presence or absence of radioactive material. An ionization type meter is necessary for accurate quantitative post‐treatment measurement of exposure rate from the microsphere vial, waste container, and patient.

Yttrium‐90 microspheres are exempt from 10 CFR 35.67(b) leakage testing requirements. Licensees should survey, with an appropriate radiation detection survey instrument (i.e., Geiger‐Muller counter or scintillation detector), all areas where the ^90^Y microspheres are prepared for use or administered, as required by 10 CFR 35.70. This includes the hot lab, procedure room, and the hands, feet, and clothing of personnel who handled the microsphere vial shields or participated in the administration. The survey should be conducted immediately following each dose preparation and administration. Survey records must be maintained as required by 10 CFR 35.2070.

Surveys in the recovery area are not necessary unless a major spill of blood or urine in that location is suspected. There is minimal urinary excretion of TheraSphere (0.011%). A small amount of ^90^Y urinary excretion (0.119%) may occur in patients treated with SIR‐Spheres. Fecal excretion is markedly less than renal excretion.^23^

### 4.4 Written directive

Each administration of ^90^Y requires a Written Directive (WD). The NRC specifies use of the following WD directive condition:

The written directive shall include the patient or human research subject's name; the treatment site; the radionuclide (including the physical form [^90^Y microspheres]); the model of spheres (e.g., TheraSphere or SIR‐Spheres) or manufacturer; the prescribed dose or activity; and, if appropriate for the type of microsphere used, the statement “or dose or activity delivered at stasis.”

The treatment site is defined in 10 CFR 35.2 as the anatomical description of the tissue intended to receive an absorbed dose. The NRC guidance document^24^ states that prescribed dose means the mean absorbed dose (rad or Gy). Alternatively, prescribed activity (mCi or GBq) may be used.

The WD may be prepared by the QMP, CHP, or RSO but the WD must be reviewed, signed, and dated by an AU before the ^90^Y microsphere administration unless a delay in order to provide a WD would jeopardize the patient's health. In addition to requiring a WD, 10 CFR 35.41 specifies other requirements to help ensure that the correct isotope, microsphere product, and dose are delivered to the intended patient and that the procedure is performed according to the WD. The licensee must retain a copy of each WD for a minimum of 3 years (10 CFR 35.2040). A copy of the licensee's procedures for Y‐90 administration must be kept for the lifetime of the license (10 CFR 35.2041).

If the administration is terminated because of stasis, the absorbed dose or administered activity to the treatment site is the value of the absorbed dose or activity administered when stasis occurred, and the administration was terminated. Stasis is defined in the NRC guidance document^24^ as a stoppage or slowdown in the flow of blood and may occur when administering a large number of microspheres. The inability to complete administration due to clogging or kinking of the catheter is not considered stasis. The record must be prepared within 24 h after the completion or termination of the administration and must include the name of the individual who determined the absorbed dose or administered activity, the signature of an AU for ^90^Y microspheres, and the date signed.

Modifications to the WD are allowed if the AU determines the procedure must be modified due to emergent patient conditions (e.g., arterial spasm or new arterial supply found). The AU must document these changes in the WD within 24 h after the completion or termination of the administration.

### 4.5 Medical event reporting rules

The licensee is required to report medical events to the NRC or Agreement State per criteria listed in their license. The NRC recommended criteria for medical event reporting are:

1. the administration of byproduct material results in a dose that exceeds 0.05 Sv (5 rem) effective dose equivalent or 0.5 Sv (50 rem) to an organ or tissue; and
2. an administration of the wrong radionuclide or type of microsphere; or
3. an administration to the wrong individual or human research subject; or
4. an administration by the wrong route of administration; or
5. an administration by the wrong mode of treatment; or
6. the total dose or activity delivered differs from the prescribed dose or activity, as documented in the written directive, by 20% or more, except when stasis or emergent patient conditions are documented and resulted in a total dose or activity administered that was less than that prescribed; or
7. a dose to the skin or an organ or tissue other than the treatment site that exceeds by 0.5 Sv (50 rem) to an organ or tissue and 50% or more of the dose expected from the administration defined in the written directive excluding shunting when it was evaluated prior to the treatment in accordance with the manufacturer's procedures.

Medical event reporting and notification requirements are given in 10 CFR 35.3045(b)–(g). Exceptions to reporting are events caused by stasis, shunting or because of patient intervention, whether intentional or unintentional, such as dislodging or removing treatment devices or prematurely terminating the administration.

### 4.6 Staff safety

The door to the procedure room must be posted with appropriate radiation warning signs. During the procedure, the primary staff safety risks involve potential contamination. Post‐procedure risks are lower and involve bremstrahhlung radiation from the patient. Nursing staff caring for the patient after the procedure should maintain a distance of at least 3 feet from the patient's liver, preferably providing all care from the patient's left side. Doses to staff caring for these patients after treatment is below public exposure limits. There is no need to exclude pregnant staff from working in these areas.

Per 10 CFR 20.1502, the NRC requires licensees to monitor occupational exposure to radiation from NRC licensed (i.e., ^90^Y and ^99m^Tc) and unlicensed radiation sources (i.e., fluoroscopy) under the control of the licensee. All staff involved in the procedure should wear appropriate personnel dosimeters. This would include whole body or collar dosimeters for all nuclear medicine, IR staff or others present in the room during administration. Nursing staff in the patient recovery area do not normally require dosimeters, although this depends on patient volume. For very busy departments, one or two nursing staff may wear dosimeters to determine if wider implementation is necessary. Extremity (ring) dosimeters should be provided for anyone handling radioactive materials or waste that may be contaminated with ^90^Y.

Proper wearing and return of the dosimeters should be monitored to ensure that the dose readings align with expected values depending on case volumes. Whole body dosimetry results are not expected to be substantially higher than equivalent roles of staff not involved in ^90^Y procedures.

## 5 PERSONNEL AND TRAINING

Personnel involved in the planning, preparation, and delivery of ^90^Y microsphere treatments must be appropriately trained. Roles and responsibilities must be defined prior to starting a ^90^Y program. For example, the facility should determine who is responsible for transporting the microspheres and who is responsible for preparing the IR suite.

Activities should be performed within the scope of the employee's training and their professional scope of practice. For example, it is appropriate for IR technologists to prime delivery device tubing and for QMPs or CHPs to survey staff and rooms for contamination. Exceptions to this can be made but more in‐depth training must be conducted when staff are operating outside of their normal work responsibilities.

Training must be conducted by qualified individuals in each specialization and, if involved in direct preparation or delivery of the ^90^Y microspheres, must include vendor training. The site must ensure that all participants are operating within their scope of practice in compliance with local regulations. The major specializations are as follows:

1. AUs;
2. NMTs;
3. Interventional radiology staff;
4. Qualified Medical Physicists.

All members of the ^90^Y microsphere team should receive annual refresher training. It is recommended that all members of the ^90^Y microsphere team receive annual refresher training about policy, equipment and software changes that affect ^90^Y microsphere treatments.

### 5.1 Overarching training

Due to the nature of the ^90^Y microsphere treatment, there are several training elements that are common to all the specializations. Radiation safety is key for the safe handling and delivery of the ^90^Y microspheres. Training must include content led by a QMP, CHP, or RSO and should cover the basics of the ^90^Y decay scheme and the differences between radiation protection for beta emitters and x‐ray sources. This should include specifics on equipment placement in the IR suite, shielding needed for the activity to be delivered, waste management, and appropriate ALARA techniques to minimize staff radiation exposure during the radioembolization procedure. This training should also include high‐level instruction on spill clean‐up and room preparation. All personnel must be aware of how a microsphere spill differs from that of a liquid solution due to the tendency of microspheres to roll over a large distance and should be knowledgeable in basic containment techniques. During training, specific responsibilities should be agreed upon and assigned to each specialization to ensure a quick and efficient clean‐up if a spill occurs. All personnel involved in the treatment delivery should be trained by the vendor in proper delivery box setup and teardown. All staff involved should complete an annual review of medical events occurring nationwide to discuss and improve clinical practice with the intent of avoiding similar occurrences.

### 5.2 AU training

To become an AU in the use of ^90^Y microspheres, a physician must satisfy the requirements put forth by the NRC's “Yttrium‐90 Microsphere Brachytherapy Sources and Devices TheraSphere and SIR‐Spheres Licensing Guidance”^24^ document or applicable Agreement State guidance. Licensees may submit alternative training and experience for approval on a case‐by‐case basis by NRC or Agreement State staff as described in Section 2.a.ii above.

The AU should also be trained in the basics of both pre‐implant and post‐implant dosimetry by a QMP. This should include ^99m^Tc‐ MAA LSF calculations, ^90^Y activity calculations, preparing the WD, volume and/or image‐based dosimetry, and post‐implant image verification.

### 5.3 NMTs

NMTs must be trained in activity preparation for the ^99m^Tc‐ MAA injections used for the calculation of the LSF. If deemed the responsibility of the NMTs, they should be trained on region selection and region of interest (ROI) creation to calculate the LSF.

For both microsphere types, the technologists must be trained by a QMP, CHP, or RSO on the correct use of the dose calibrator when measuring the activity of the ^90^Y vial. It is understood that the QMP, CHP, or RSO are operating under the overarching authority of the AU. For SIR‐Spheres specifically, the technologists must be trained in the proper dose dispensing protocol and radioactive spill safety and cleanup. This training should be conducted by the vendor.

### 5.4 IR staff

#### 5.4.1 IR physicians (non‐AU)

If the IR physician desires to become an AU, the AU qualification training in section 3.B must be completed. Regardless of AU status, training for all IR physicians must include:

1. the set‐up and disassembly of the delivery device;
2. the minimum required catheter inner diameters (ID ≥ 0.5 mm / 0.020 inch for TheraSphere and ID ≥ 0.8 mm / 0.031 inch for SIR‐Spheres) and
3. the basics of both pre‐implant dosimetry and post‐implant treatment delivery verification.

The vendor should provide training for (1) and (2). A QMP, CHP, or RSO, should provide training on (3). IR physicians should have a basic understanding of staff roles in the IR suite in case of an emergency or radioactive spill. The IR physician should have instruction in radiation protection and written directive procedures and license conditions associated with the microspheres as required per 10 CFR 35.27. As with AUs, the IR physician should participate in annual reviews of reported medical events.

#### 5.4.2 IR technologists and staff

All technologists, nurses, and other staff who will be in the procedure room during ^90^Y microsphere administration must have basic training in radiation safety. Training should include information specific to beta radiation and ^90^Y such as range in air and tissue and fluoroscopy radiation safety. IR technologists are most commonly assigned the tasks of preparing the room for the procedure. They must be trained on proper delivery device setup typically by the vendor, QMP, CHP, or RSO. If the technologist is not responsible, then the personnel responsible should be trained. Regardless of their role, all staff present in the IR room during administration of the ^90^Y microspheres must be trained in radioactive spill response, which includes being surveyed and cleared by radiation safety staff before exiting the room.

### 5.5 RSOs and QMPs

Requirements put forth by the NRC's “Yttrium‐90 Microsphere Brachytherapy Sources and Devices TheraSphere and SIR‐Spheres Licensing Guidance” document in reference to 10 CFR 35 state that the RSO must have training specified in 10 CFR 35.50. This training should include all steps of the ^90^Y procedure from activity arrival and package receipt to waste disposal and clean up. An emphasis must be placed on pre‐treatment dosimetry and post‐treatment verification and dosimetry, radiation safety, regulatory issues, and emergency procedures for ^90^Y use.^25^ Training should be provided by the applicable vendor(s) and additional training given by an experienced QMP, CHP, or RSO. Training must include specific license conditions and practical operational issues that may not be covered by regulatory requirements. For both microsphere types, the QMP, CHP, or RSO must be trained in the correct use of the dose calibrator when measuring the activity of the ^90^Y vial.

## 6 PRE‐TREATMENT IMAGING

Imaging procedures are a core workup component for TARE. Each patient has a unique arterial supply that determines the distribution of the injected ^90^Y microspheres. Imaging is essential to map a patient's hepatic vasculature and finalize patient eligibility for treatment.

### 6.1 Angiography work‐up, extra‐hepatic embolization

Angiography is performed to map the patient's hepatic artery architecture. The purpose of arterial mapping in TARE is both diagnostic (determining the hepatic and tumor vascular supply and calculating the volumes of treated regions) and for interventional guidance (to guide embolization of variant mesenteric supply to prevent non‐target irradiation and calculate volumes of treated regions).^26^ DSA has been the most common angiography method but cone beam computed tomography (CBCT) has surpassed DSA in effectively identifying hepatic vessels for embolization and TARE. It has been shown that CBCT can identify tumors, supplying vessels, and extra‐hepatic vessels more reliably than planar imaging^27^ and has been used to calculate the volume of the area to be treated and tumor volume.^28^ CBCT is an important adjunct to DSA and mapping studies should be performed on CBCT‐capable equipment when possible. The interventional radiologist should be familiar with how to perform CBCT in the angiography suite, and the facility should ensure that appropriate CBCT protocols are available.

The angiography procedure can be performed under moderate sedation or general anesthesia through femoral or radial artery access. The following is a description of a typical treatment workflow, but the specifics will vary. These decisions are the domain of the interventional radiologist administering the treatment. A sheath is inserted and an angiography catheter is advanced into to the superior mesenteric artery (SMA).^26^, ^29^ Angiography rules out replaced or accessory hepatic arterial supply. The celiac artery is then selected with angiography to determine hepatic arterial supply and vascular flow dynamics. Vascular flow may be altered by certain systemic therapies such as bevacizumab, which has been associated with stasis and ulcers.^30^ Using a co‐axial method, a microcatheter and microwire are advanced, with selective catheterization of the proper, right, left, or segmental hepatic arteries depending on the treatment goal.^26^, ^29^ An angiogram must be obtained from the planned treatment location. Physicists unfamiliar with these images should consult the article by Roncali et al.^31^ for example, angiograms. Most commonly, ^99m^Tc macroaggregated albumin (^99m^Tc‐MAA) is then administered.

In addition to determining tumor vascular supply, the mapping procedure is performed to eliminate any mesenteric vascular supply. Alternatively, mesenteric supply may be eliminated on the day of treatment prior to ^90^Y administration. Delivery of ^90^Y microspheres proximal to vessels supplying the gastro‐intestinal (GI) tract will result in radiation ulcers. Therefore, it is important to embolize any vessels supplying the GI tract emanating from the hepatic arterial tree distal to the proposed administration site. Examples include right gastric, supra‐duodenal, esophageal, and accessory gastric arteries.^26^, ^29^ Vessels whose origin is in the vicinity of up to 2 cm proximal to the catheter location may need to be embolized depending on flow, volume of microspheres, and size of the vessel. Systematic embolization of the gastroduodenal artery (GDA) is no longer performed.

Before the end of the mapping procedure, ^99m^Tc‐MAA is administered. A single ^99m^Tc‐MAA injection is used for both assessment of LSF and the distribution within the liver. Upon completion of the mapping procedure in IR, the patient must be imaged in nuclear medicine for pre‐treatment planning. Planar or SPECT/CT imaging is used to determine the distribution of ^99m^Tc‐MAA particles. Technetium‐99m MAA and ^90^Y have similar biodistributions, so the ^99m^Tc‐MAA imaging is a reasonable predictor of ^90^Y dose uptake and distribution during treatment. The agreement between imaged ^99m^Tc‐MAA and actual ^90^Y distributions depends on the imaging and dosimetric methods used. With the exception of the body surface area (BSA) method, ^99m^Tc‐MAA imaging data will be combined with volumetric data from CT or MRI to calculate the ^90^Y microsphere activity to administer, based on a prescribed absorbed dose to a target region. The target volume is often either a liver lobe or a liver segment/partial segment. SPECT/CT is more versatile than planar imaging for pre‐treatment planning, as SPECT/CT provides information regarding the fraction of activity within sub‐regions of the treated liver. Specifically, with SPECT/CT tumor and normal tissue can be delineated. This allows the projected microsphere activity to be quantified in both regions, ultimately allowing activity prescriptions based on lesion target as well as healthy liver tissue absorbed dose constraints. There is evidence of an overall survival benefit when prescribing administered activity based on absorbed dose to the tumor while applying healthy liver dose constraints versus the standard whole treated area (lobe) of liver for HCC patients.^32^ In the first step of pre‐treatment planning, ^99m^Tc‐MAA planar images must be obtained. If available, SPECT/CT imaging should be subsequently performed.

### 6.2 Technetium‐99 m MAA handling, preparation, and administration

Technetium‐99m MAA is a radiopharmaceutical composed of a macroaggregate of human serum albumin bound to ^99m^Tc. Particle size distributions may vary with kit manufacturer, preparation technique, and time from preparation, but must have at least 90% of particles between 10 and 90 μm.^33^ Technetium‐99m MAA should be used within approximately 4 h of preparation to ensure appropriate particle size. While this is a larger range of particle sizes than found with currently commercially available microsphere products (see Table 1 above), ^99m^Tc‐MAA planar imaging has remained the most common technique in pre‐treatment planning for calculating the LSF. The LSF is the fraction of activity administered to the patient that leaks from the liver or tumors, returns to the circulatory system, and embolizes in the lungs.

Technetium‐99m MAA is commonly used for lung perfusion imaging during nuclear medicine lung ventilation/perfusion (V/Q) scans. While most nuclear medicine departments and technologists are familiar with the special considerations MAA requires, consideration should be given to radioactive material use and safe handling in a department outside of nuclear medicine. If the interventional radiologist prefers to transfer the radioactive material from the syringe provided by nuclear medicine, the transfer should be done above disposable absorbent pads. The syringe and gloves of anyone handling radioactive material must be returned to nuclear medicine or stored and disposed of per site RAM license requirements. The room and staff must be surveyed for radionuclide contamination.

### 6.3 The LSF

The LSF is a measure of the proportion of the ^99m^Tc‐MAA that passes through arteriovenous connections, thereby bypassing the liver parenchyma and lodging in the pulmonary capillary bed. Arteriovenous shunting can be higher than usual in cirrhotic livers and within tumorous tissue. The LSF enables more accurate estimation of the amount of administered ^90^Y‐microspheres that will lodge in the target tissue as well as estimation of radiation pneumonitis risk. Different calculation methodologies have been used to determine LSF, but all techniques generally reflect some measure of lung counts/(lung counts + liver counts).

LSFs must be calculated. LSF determination should be based on planar imaging unless an institution has validated LSF calculations from SPECT data. Most historical data related to lung toxicity has been obtained from planar images. Studies have shown that planar imaging tends to overestimate the LSF.^19^ More accurate LSFs may be calculated with SPECT/CT but will generally require at least two bed positions of imaging. SPECT/CT‐based ^99m^Tc‐MAA imaging will also provide more accurate dosimetric estimates. Any quantitative dosimetry should be calculated from SPECT/CT images.

LSF safety thresholds have historically been derived from planar images without any attenuation corrections.^34^ However, the lungs are better visualized on a posterior view because of reduced cardiac attenuation. The liver is better visualized on an anterior view because of its more anterior position.^35^ Planar LSFs should be calculated using formula (1) below, with $Counts_{Liver}^{Background}$ measured in a small ROI just inferior to the liver and $Counts_{Lung}^{Background}$ measured laterally from the apices of the lungs. Geometric mean of the planar anterior and posterior images may also be used.

(1) $$LSF\;=\frac{Counts_{Lung}^{Posterior}\;-Counts_{Lung}^{Background}}{\left(Counts_{Lung}^{Posterior}\;-\;Counts_{Lung}^{Background}\right)+\;\left(Counts_{Liver}^{Anterior}\;-\;Counts_{Liver}^{Background}\right)}\;$$

For sites using SPECT/CT, the LSF should be calculated using Equation (1) but using counts from volumes of interest rather than views and with no background subtraction. When SPECT/CT is used, attenuation correction and scatter correction, such as triple energy window scatter correction, provide more accurate LSFs and should be used.^35^ Reconstruction parameters should be matched between bed positions whenever multiple SPECT data sets are acquired. Details on SPECT reconstruction parameters are outlined in Kunnen et al.^36^

A whole lung absorbed dose limit of 30 Gy is typically used for a single administration, with a total maximum lung absorbed dose of 50 Gy from multiple administrations. Before proceeding with treatment, the AU and a QMP must discuss possible causes and changes to treatment for LSFs exceeding 20% or lung doses over 30 Gy. The same process should be used for LSFs between 10% and 20%. These absorbed doses and LSF percent limits have historically been calculated with ^99m^Tc‐MAA planar imaging and should be calculated in the same fashion when using these limits unless SPECT‐based LSF methods have been validated. Validation could consist of calculating LSF using planar and SPECT for a number of patients until differences between the calculated values are well understood.

Factors affecting the LSF calculation include treatment location and lobar versus segmental approaches, time from ^99m^Tc‐MAA preparation to administration, and time from ^99m^Tc‐MAA administration to imaging. In general, nuclear medicine imaging should be performed as soon as possible after ^99m^Tc‐MAA administration. Prolonged delays greater than a few hours between ^99m^Tc‐MAA administration and imaging produces an elevated LSF,^37^ due to the presence of increased ‘free’ pertechnetate. LSF values will also depend on patient and tumor characteristics including primary tumor type, lesion size, and chemotherapeutic history. A higher LSF may correlate with decreased overall survival in patients with liver‐dominant metastatic disease.^38^ Parallel to mapping procedures, LSF should be repeated if there is reason to believe the liver or tumor vasculature has changed or if the prior LSF is more than 1 year old, with additional consideration given in cases where the initial LSF exceeds 5%.

When selecting where to inject ^99m^Tc‐MAA, the physician must weigh several considerations: administering the entire activity to the lobe or treatment location suspected to have the largest LSF may represent a worst‐case scenario but could miss smaller shunts not visible on angiograms or CBCT. Delivering the ^99m^Tc‐MAA fractionally at each treatment location within the liver may represent a compromise in assessing the LSF globally while potentially underestimating the LSF at any one treatment site. With replaced arteries, the ^99m^Tc‐MAA may need to be split into separate injections. Regardless, ^99m^Tc‐MAA should be administered from the same territory as the ^90^Y planned administration. In general, the most clinically relevant LSFs will be calculated when ^99m^Tc‐MAA is administered singly at the same location as the anticipated treatment. This would necessitate remapping the patient before each treatment.

## 7 TREATMENT PLANNING

### 7.1 Dosimetry treatment plan

The goal of pre‐treatment dosimetry is to provide projected ^90^Y‐microsphere absorbed dose calculations [Gy] to regions based on the ^99m^Tc‐MAA distributions. These will determine the quantity of activity to administer based on target objectives as well as normal tissue constraints.

Treatment planning for radioembolization dosimetry can be described in the following generalized steps:

1. A surrogate radiopharmaceutical (e.g., ^99m^Tc‐MAA) is injected and imaged using planar or SPECT/CT nuclear medicine techniques;
2. The LSF is calculated based on the surrogate radiopharmaceutical imaging as discussed in Section 4 above;
3. Target absorbed dose is prescribed. This is typically based on treatment efficacy goals or absorbed dose constraints to normal organs;
4. Prescribed activity is calculated based on the absorbed dose goals and constraints set in step 3;
5. The absorbed dose to the lungs and other non‐target organs at risk with activity depositions is calculated based on the prescribed activity. If absorbed dose values to non‐target organs exceed the targets in step 3, adjust prescribed activity in step 4 and repeat.

### 7.2 General activity and absorbed dose formalisms

Except for the SIR‐Spheres BSA method (discussed later), treatment planning steps require a dosimetric method for converting between measured pre‐therapeutic (^99m^Tc‐MAA surrogate) activity and prescribed or predicted therapeutic (^90^Y‐microsphere) absorbed dose. For the purposes of dosimetry, microspheres may be considered radiopharmaceuticals. However, historically, the modality has evolved separately and developed its own nomenclature, often at odds with standard radiopharmaceutical dosimetric terminology. In particular, the “Medical Internal Radiation Dose (MIRD) model” is often used to refer exclusively to the mono‐compartmental or whole organ approach. Nearly all the dosimetric approaches for microspheres or “models” (mono‐compartmental model, partition model, multi‐compartmental model, etc.)^1^, ^39^ are derived from, or can be reduced to, the MIRD method, also known as the absorbed fraction method. The exceptions are Monte Carlo or dose‐kernel‐based simulation methods^a^.

The MIRD method, in its most general form, considers the mean absorbed dose, *D*, to a target region, *r_T_*, as the sum of all the absorbed dose contributions from activity in all the potential source regions, *r_S_*, where the activity, *A*, is assumed uniform in each individual source region.^40^

The MIRD formalism applied to ^99m^Tc‐MAA/^90^Y‐microsphere dosimetry benefits from two major simplifying assumptions:

1. Radioembolization implies that the activity does not re‐distribute as a function of time. Therefore, the activity as a function of time is a mono‐exponential characterized by the ^90^Y physical decay constant, λ_Y90_:
(2) $$A\left(r_{s},t\right)=A\left(r_{s},0\right)e^{-\lambda_{Y90}*t}$$
  where *A(r_s_,0)* is the activity in the source region at time of administration (*t* = 0). Consequently:
(3) $$\tilde{A}\left(r_{s}\right)=\frac{A\left(r_{s},0\right)}{\lambda_{Y90}}$$
$\tilde{A}\left(r_{s}\right)$ is the integral of the activity, known as the time‐integrated activity (TIA). It represents the total number of decays in a source region.
2. Yttrium‐90 is a nearly pure beta‐emitter, with a very low branching ratio of prompt gammas and some Bremsstrahlung photons. It is reasonable to assume that all energy from the decay is deposited locally.^41^, ^42^, ^43^ Since the mean energy per disintegration, E, is constant the activity‐to‐energy conversion factor, k, is the same for all sources and targets. Henceforth, *ROI = r_S_ = r_T_*.
(4) $$k=\bar{E}*\frac{1}{\lambda_{Y90}}$$
  Given E = 0.9267 MeV per disintegration, k is represented numerically as:
(5) $$\begin{array}{ccc} 49.38\;\left[\frac{J}{GBq}\right]\; & = & 0.9267\;\left[\frac{MeV}{dis}\right]*\frac{2.6684}{\ln\left(2\right)}\left[day\right]*\\ & & \;\;\;\;\;\frac{86400}{1}\left[\frac{s}{day}\right]*\frac{10^{9}}{1\;}\left[\frac{Bq}{GBq}\right]*\frac{1\frac{dis}{s}}{Bq}*\\ & & \;\;\;\;\;1.6022x10^{-13}\left[\frac{J}{MeV}\right] \end{array}$$
  The average energy of ^90^Y decay exists within the literature as several different values due to minor variations in the number of significant figures used in the activity‐to‐energy conversion constant *k*.
3. In addition to these two simplifications, because of the lack of pharmacokinetics, and the limited number of organs where the activity is expected to localize, relative rather than absolute dosimetry is generally used. It is assumed that microspheres are deposited solely within the liver and the lungs for purposes of quantification. This reduces to determining the fraction of activity, *f_ROI_*, of the entire administered activity, *A_0_*, in a region equal to the fraction of counts present in the region derived from either ^99m^Tc‐MAA planar or SPECT/CT images.
(6) $$f_{ROI}=\frac{c_{ROI}\left[counts\right]}{\sum_{1}^{n}c_{ROI}\left[counts\right]}$$

Equation (6) equates to the LSF equation when the denominator consists of *c_lungs_* and *c_liver_* and the numerator is *c_lungs_*_.,_ that is, *f_lungs_* = LSF.

The activity in the ROI, *A_ROI_*, is thus related to the administered activity, *A_0_*, by the fraction of counts present in the region derived from either ^99m^Tc‐MAA planar or SPECT images, *f_ROI_*.

(7) $$A_{ROI}=f_{ROI}*A_{0}$$

These three simplifications, combined, mean that fractional activity measured in any defined ROI at any time point on the pre‐therapeutic ^99m^Tc‐MAA image is expected to be equal to the fractional activity and fractional absorbed dose at every time point for the ^90^Y‐microsphere treatment for the same region. Substituting these into the MIRD formalism results:

(8) $$D_{ROI}\left[Gy\right]=\frac{49.38\;\left[\frac{J}{GBq}\right]*A_{0}\left[GBq\right]}{M_{ROI}\left[kg\right]}*f_{ROI}$$

where the mean absorbed dose to any ROI from ^90^Y‐microsphere therapy is related to the administered activity A_0_ and is proportional to the fraction of activity in the ROI as measured by the pre‐therapeutic ^99m^Tc‐MAA.

Equation (8) is a formula, that is valid for all approaches, including the mono‐compartmental, partition, and voxel‐based local deposition methods (See Section 5.C.) and can be used to find the absorbed dose for all ROIs: liver, lobe, lung, individual tumor, collective tumors, normal tissue, voxel, and so forth. Minor variations of this formula exist in the literature:

1. If the activity distribution in the pre‐therapeutic scenario is characterized by a single treated liver volume and the LSF, *F*, then the fractions of activity for the treated portion of the liver, *f_liver_*, and the lungs, *f_lungs_*, are *(1‐F)* and *F*, respectively.
2. The volumes of different ROIs such as liver tissue or lesions may be determined by many modalities. Conversion from volume to mass is variable in the literature. Volume to mass conversions should use 1.03 g/cm^3^ for the liver. A soft tissue density of 1.03 g/cm^3^ should be used for organs where mass densities are not available.
3. A density of 0.26 g/cm^3^ is a reasonable average lung density for the patient population typically receiving TARE.^44^, ^45^, ^46^ The density may change with age and respiratory phase during acquisition. Patient specific lung mass estimation techniques exist. These may be used when lung absorbed dose is a limiting factor.^47^ Alternatively, a standard reference lung mass of 1 kg is often used. This is done when there is no 3D imaging information for the lung and the lung mass is unknown. The 1 kg mass is derived from a historical reference lung mass from the International Commission on Radiation Protection (ICRP) Publication 23^48^ for a 70 kg male. ICRP Publication 89^49^ updated that value to 1.2 kg for men and 0.95 kg for women.

Activity is assumed to be localized within the liver and lungs. This is a reasonable assumption, but a qualitative check of other regions of the body should be made on SPECT. If activity was deposited outside the liver and lungs, these regions must be included in the denominator of Equation (6), but more generally may represent a contraindication to proceeding with the procedure. When only planar images are used, the activity is assumed to be completely contained within the liver and lungs. Regardless of the imaging modality used, dosimetry must be performed after pre‐treatment ^99m^Tc‐MAA imaging, unless the BSA model (discussed below) is used.

In the last decade, Monte Carlo based dosimetric methods have been introduced, which also include dose kernel convolution methods for converting activity to absorbed dose. This is an active area of investigation, both for microspheres and for radiopharmaceuticals in general.^50^, ^51^ In radiopharmaceutical therapy (RPT), these are often called “voxelized” methodologies and use patient‐specific anatomy for simulation of decay, radiation transport, and fractionalized energy depositions. This is in contrast to the MIRD method, which for most RPTs requires S values taken from anthropomorphic phantom models and only collects TIA in the different organs. The approximation of localized energy deposition for ^90^Y means that the MIRD methodology as outlined above can be used with the patient‐specific anatomy for dosimetry at any level down to the individual voxels. In theory, Monte Carlo‐based methods are superior as they simulate the radiation transport of decayed particles. However, current clinical imaging modalities have an imprecision in the localization of measured activity, known as spill‐out or partial volume effect, on the order of 3–5 mm.^41^, ^42^, ^43^ This uncertainty in localization is similar in magnitude to the range over which the absorbed dose from ^90^Y decay is deposited, such that, the uncertainty in measurement mimics the energy deposition pattern from radiation transport to a reasonable degree. For most RPTs, Monte Carlo‐based methodologies are more accurate than localized energy deposition methods. It is unclear whether this is the case for ^90^Y microspheres.

### 7.3 Administered activity calculations for TheraSphere and SIR‐spheres

TheraSphere and SIR‐Spheres provide package inserts with instructions to perform activity or absorbed dose calculations.

TheraSphere follows the five steps of the treatment plan outlined in Section 5.A. Dosimetry follows the MIRD equation in Equation (8). Re‐writing this formula to solve for administered activity based on a target absorbed dose becomes:

(9) $$A_{0}\left[GBq\right]=\frac{D_{ROI}\left[Gy\right]*M_{ROI}\left[kg\right]}{49.38\;\left[\frac{J}{GBq}\right]*f_{ROI}}$$

Equation (9) provides flexibility in obtaining a prescribed activity dependent on a prescribed absorbed dose. For TheraSphere, the indicated dosimetric endpoint presented in the package insert is presumed to be a lobar or whole liver absorbed dose, which includes both normal tissue and disease, in the range of 80 – 150 Gy; typically, 120 Gy to the treated liver volume, *M_liver_*.

However, two alternative targeting strategies may be employed:

1. At the discretion of the AU, administered activity may be calculated using a lobar volume but delivered segmentally, with the intention of completely ablating a single segment of the liver. Dosimetry in this case should be updated to calculate the segmental absorbed dose.
2. Another endpoint is to determine a maximum allowed absorbed dose to normal liver *D*_*normal*_ to both spare healthy tissue from excessive irradiation and allow for higher absorbed dose delivery to the lesion(s). Target lesion absorbed dose(s), *D*_*tumor*_ may be used in combination with threshold absorbed dose. This delineation between normal tissue and lesion(s) is known as the partition, or multi‐compartmental model. Lesions may be considered individually or as a whole. ^52^ While Equation (9) is recommended for this approach, a separate mathematical formalism, based on the ratio between uptake in tumor versus uptake in normal tissue, has been developed for this approach^52^, ^53^, ^54^, ^55^ and is often used.

In each of these approaches, the value of *D_ROI_*, which represents a mean absorbed dose to a specified ROI (i.e., liver) may be changed to find a new prescribed activity. This new prescribed activity should then be back‐substituted into Equation (8) to recalculate the absorbed dose in other ROIs (i.e., lung) as listed in Step 4 of Section 5.1. *D_ROI_* may be set for only one region at a time.

In contrast, SIR‐Spheres has two mathematical models, plus a historic method which is no longer recommended, known as the empiric method. Of the two methods currently in use, the BSA model does not follow MIRD formalisms nor apply any dosimetric principles. This methodology skips steps 3 and 5 in the pre‐treatment planning and calculates the prescribed activity solely based on volumetric data. Imaging with ^99m^Tc‐MAA is not used directly to perform dosimetry, but rather used to heuristically modify the prescribed activity.

The BSA is the most widely used treatment planning technique for SIR‐spheres. It is an empirically established formula,^44^ dependent on the patient's height, weight, and relative tumor burden.

(10) $$BSA\;=\;0.20247*H^{0.725}*W^{0.425}$$

where *H* is the patient height in meters and *W* is patient weight in kilograms. Nominally, the units of BSA are *m^2^*, although the formula as generally presented is inconsistent with regard to units. The administered activity, *A_0_*, for a whole liver treatment, is then given by:

(11) $$A_{0}\left[GBq\right]=\left(BSA-0.2\left[\frac{V_{tumor}}{V_{tumor}+V_{normal}}\right]\right)$$

where *V_tumor_* is tumor volume and *V_normal_* is normal liver tissue volume. The limitation of this method lies in assuming a positive relationship between the patient's physical size and ability to tolerate higher activity.^56^

For lobar and segmental therapy, only portions of the liver are being treated and Equation (11) must be modified by the ratio of targeted volume to whole liver volume:

(12) $$A_{0}\left[GBq\right]=\left(BSA-0.2\left[\frac{V_{tumor}}{V_{tumor}+V_{normal}}\right]\left[\frac{V_{target}}{V_{Liver}}\right]\right)$$

Although a prescribed absorbed dose (Step 3) is not used to calculate the prescribed activity (Step 4), planar or SPECT imaging is still performed. Therefore, absorbed dose estimates are possible and should be calculated with Equation (8) (Step 5).

### 7.4 Segmentation

Liver tissue volumes can be obtained in several ways, but is usually done using segmentation software. Often a contrast enhanced CT exam is imported into the software and used to delineate whole liver volume, treatment volumes (i.e., for lobar treatments, the right and left hepatic lobes) and tumors within treatment volumes. Classically, the whole liver is divided into the left and right lobes by the middle hepatic vein (MHV). Alternatively, the use of in‐suite CBCT during pre‐treatment angiography enables the interventional radiologist to visualize, in real time, the perfused volumes fed by the left and right hepatic arteries delineating the corresponding lobes.

For tumor delineation, a site must choose between anatomical versus functional imaging. Anatomical techniques like contrast‐enhanced CT or MRI produce lower contrast between tumor and healthy liver tissue than functional imaging but allow accurate geometric volumes once the tumor margins are identified. Functional imaging modalities like SPECT or PET provide higher inherent contrast but at lower resolution and can miss necrotic or other low uptake regions. CBCT can be considered a dual function modality that can provide both functional and anatomical information. CBCT is considered functional imaging when revealing what tissue is perfused by a given artery as well as anatomical imaging when determining the hepatic lobes. Anatomical imaging for segmentation is recommended unless functional imaging is used in conjunction with anatomical imaging (i.e., SPECT/CT, PET/CT, or PET/MRI).

Several software packages are commercially available for segmentation, enabling clinicians to use time‐saving tools such as automated, or semi‐automated, segmentation. These include image registration tools that take advantage of multiple imaging studies the patient may have undergone in preparation for liver radioembolization. Software packages for performing manual segmentation are also available, albeit these are more time‐consuming to use.

### 7.5 Report contents

Physicists should prepare a report documenting the imaging and dosimetry methods used for pre‐treatment dosimetry as applicable. Examples of the content to include are summarized in Table 3.

**TABLE 3:** Suggested report contents.

Entry	Parameters	Example
Radiopharmaceutical	^99m^Tc‐MAA, ^90^Y, other	^99m^Tc‐MAA
Imaging modality	Planar, SPECT/CT, PET/CT, other	Planar
Reconstruction	**If Planar**: geometric mean, posterior, anterior, other	Anterior and posterior views
	**If SPECT/CT or PET/CT (Software)**: Iteration, subsets, corrections, full width half maximum (FWHM), other	SPECT/CT with attenuation correction, collimator detector response correction, empirical scatter correction, no filter with in‐house software. CT was calculated with filtered back projection
Segmentation	**Modality**: Planar, CT, MRI, CBCT, SPECT/CT, PET/CT	Planar, CT
	**Drawn ROIs (Software)**: Liver, Lungs, other	Segmented liver on anterior view, lung on posterior view Segmented liver and lungs on CT
Dosimetry	**Method (Software / Deposition Model)**	MIRD calculated by hand. SPECT/CT with in‐house voxel‐based MIRD software.
	**Mass Determination**: CT, nominal, phantom	Nominal: 1.03 g/cm^3^ for soft tissue, 1.03 g/cm^3^ for liver, 0.26 g/cm^3^ for lungs
	**Calculated Regions**: Liver, lungs, other	Liver

### 7.6 Radiobiology

The absorbed dose from ^90^Y microspheres is not biologically equivalent to the same dose delivered by external beam radiation therapy. Not only are the doses delivered at different rates, but embolization using microspheres results in a small‐scale distribution of activity that is not uniform but follows a statistical pattern, with an absorbed dose distribution that varies from lobule to lobule and within each lobule. The relationship between the macroscopic absorbed dose and the micro‐scale localization has been described by anatomical modeling of individual lobules, statistical distributions of activity in lobules, and a normal tissue complication probability model.^57^ Quantities such as the biologically effective dose (BED) calculated using conventional formulations will not provide meaningful values.

## 8 DOSAGE PREPARATION AND ADMINISTRATION

### 8.1 Traceability

A QMP or Nuclear Pharmacist should verify documentation from the company that describes calibration and standard traceability of the product. Manufacturers who produce radioactive microspheres should provide and maintain standards for primary validation and measurement to the National Institute of Standards and Technology (NIST) biennially. Traceability of ^90^Y is an area of active investigation.

For SIR‐Spheres, traceability has been established through international collaborations with standards laboratories. Mo et al.^58^ established primary traceability with NIST and secondary traceability with the Australian Nuclear Science and Technology Organization (ANSTO) Radiopharmaceuticals and Industrials (ARI).

1. They sent ^90^Y solution standards to NIST and ANSTO/ARI and repeated the measurements with a different primary laboratory, Council for Scientific and Industrial Research‐National Measurement Laboratory in South Africa. The measurements between these laboratories agreed within 0.15%.
2. Once secondary traceability was established at ANSTO/ARI, they measured the activity of a SIR‐Spheres vial (non‐digested, intact spheres). Then they chemically digested the ^90^Y SIR‐Spheres and used the secondary calibration factor for the ^90^Y solution with this chemically digested solution of ^90^Y SIR‐Spheres. These two sets of measurements were used to derive a cross‐calibration factor for the intact SIR‐Spheres. A similar experiment was completed by Lourenço et al. at Laboratoire Nationale Henri Becquerel in France.^59^
3. This cross‐calibration factor at SIR‐Spheres manufacturing facilities is verified every 6 months to be within 5% of the original ANSTO calibration. Upon request from the institution, Sirtex can provide documentation of their measured activity for each vial and recommends that institutions verify their dose calibrator settings with 3 sources annually.

For TheraSphere (Boston Scientific), traceability and quantification of ^90^Y has been established with NIST. The manufacturer measures and sends 3 and 20 GBq vials to NIST for measurement of both activity and impurity profiles on a biennial basis. The manufacturer then verifies that their activity measurement equipment is operating within 2% of the NIST measurements. When manufacturing the dose vials, TheraSphere is dispensed by weight and then the activity is assayed. If the measured activity is within 10% of the nominal activity, it may be released for shipment to the institution.

### 8.2 Initial site calibration

The appropriate dose calibrator dial setting must be determined by a QMP or Nuclear Pharmacist with a vendor‐supplied product‐specific activity standard, measurement method, and source geometry. A certificate of measurement should be obtained from the manufacturer when initially receiving activity. The activity specified in the certificate should be used to determine the dose calibrator‐specific settings. Dose calibrator configuration must be performed in a manner consistent with the technique for clinical measurement. In the case of TheraSphere, this means establishing a dose calibrator dial setting with the activity contained within the glass v‐vial inside an acrylic shield. For SIR‐Spheres, a decision must be made as to whether the spheres will be assayed in suspension (immediately after agitation) or with the spheres completely settled to the bottom of the ∼20 mL glass vial (> 2 min after agitation). The latter may be more reproducible; however, the former may be clinically relevant.

At least one dose calibrator must be calibrated for ^90^Y microsphere assay. Two dose calibrators are preferred in the event of one malfunctioning or being unavailable. Dose calibrator settings will not be the same for TheraSphere and SIR‐Spheres due to differences in geometry. If an institution is using more than one dose calibrator, the dial settings may not be equivalent, even for identical dose calibrator models. The institution should establish unique settings for each device to provide the most accurate activity reading, which should be verified annually. Dose calibrator quality control (QC) must be maintained consistent with regulatory and RAM license requirements and should be consistent with AAPM TG‐181.^60^

### 8.3 Activity receipt and measurements

Personnel should avoid handling ^90^Y vials directly. Appropriate distance and shielding, such as long forceps or 1.1 cm high density polyethylene (HDPE) or acrylic shields, should be used to reduce personnel exposure.

Manufacturers should provide doses with activities within 5% of the customer's ordered value, although as noted above, the manufacturer's internal acceptable precision may be within 10%. Manufacturers must provide the factory‐assayed activity for each vial shipped. The activity for each unit must be assayed using the same methodology, geometry and setting as for the standard, as specified above. The QMP, CHP, RSO, or other radiation staff member should verify that this measured activity is within institutional tolerance guidelines (e.g., 5%) of the manufacturer's stated value and the ordered value. If the measurement is not within tolerance, the site should work with the manufacturer to determine the source of discrepancy. If a variance of more than 10% cannot be resolved the vial must not be used. The measured and manufacturer‐stated activities must be compared to the prescribed activity. Differences between the measured versus prescribed activities and manufacturer‐stated versus prescribed activities should not be greater than 5%. To reduce the possibility of a medical event, discrepancies larger than 5% should be discussed with the prescribing AU before treatment, keeping in mind that there is always some residual after administration and that ^90^Y activity decays ∼1% per hour.

Activity must be documented, labeled, and entered in the site inventory in a manner consistent with the site‐specific RAM license requirements.

### 8.4 Dosage preparation

TheraSphere vials are available in 0.5 GBq increments ranging from 3 to 20 GBq, calibrated to Sundays at 12 p.m. U.S. Eastern Time (ET). Users select the vials that, when decay corrected to the time of treatment, match the required activity for treatment. Before treatment, this vial must be assayed in a dose calibrator, as described above.

SIR‐Spheres are typically delivered at fixed activity levels, so the dispensing NMT operating under the supervision of a QMP, CHP, RSO, other radiation staff member, or Nuclear Pharmacist must draw the required activity, using aseptic technique per USP 825.^61^ The drawn activity to be administered is determined by subtraction of the remaining vial activity from the initial activity, not measured directly, and is decay corrected to the time of treatment. Vials are always calibrated for 6 pm ET. Sirtex follows daylight savings time in North America. Once drawn, the remaining activity in the vial is measured and subtracted from the initial measurement. This difference in initial versus post‐draw measurements, decay corrected to expected time of administration, should be within 5 % of the prescribed activity and must not exceed 10%. The dose calibrator setting for SIR‐Spheres has been shown to vary with volume.^53^ There is currently no clinical consensus on how to address this, but investigations are ongoing.

The vendor offers post‐day, same‐day or 1‐, 2‐, or 3‐day pre‐calibration date options where the calibration date of the standard 3 GBq vials is calibrated to same‐day of treatment or to 1‐, 2‐, or 3‐ days later. The 1‐, 2‐, or 3‐day pre‐calibration options are higher activity vials, which results in a smaller number of microspheres for a given activity at time of treatment compared to the same‐day calibration. The vendor's motivation is to not only provide more flexibility with the amount of activity available for a draw, but to also reduce the potential for stasis, though the latter has yet to be proven clinically.

### 8.5 Dosage administration

The written directive must be reviewed by two qualified individuals before transport of the activity to the IR suite. Prior to administration, the written directive must be verified and signed by the AU, and must include patient identification, treatment site, route of administration, activity, and, if applicable, absorbed dose. The prescribed activity must be compared against the patient‐specific activity vial. A time‐out with the QMP, CHP, RSO, or other radiation staff member and the treating physician(s) must be performed prior to administration. This time‐out must include all items required to be in the written directive and should include LSF and vial identification number and lot number (if applicable). These last two items are especially crucial at high‐volume treatment centers where patients’ vials could easily be mixed up with others or within a multi‐vial treatment. For multi‐vial treatments, the time‐out should specifically cover each vial's intended treatment site (target).

#### 8.5.1 SIR‐Spheres

Delivery of SIR‐Spheres must be performed using one of the two manufacturer‐provided delivery systems according to manufacturer directions.^44^, ^62^ SIR‐Spheres must be administered no more than 24 hours after the manufacturer's calibration time stamp. Ordered activity generally arrives at the institution the same day or 1–3 days prior to the calibration date/time, which may or may not be the date of administration. SIR‐Spheres must be administered using a manufacturer provided delivery apparatus, according to manufacturer directions, with a sterile 5% dextrose solution.

#### 8.5.2 TheraSphere

Delivery of TheraSphere must be performed directly from the manufacturer‐provided activity volume using a manufacturer‐provided delivery apparatus, according to manufacturer directions.^63^ TheraSphere must be administered no more than 12 days after the manufacturer's calibration time stamp, which is always Sunday at noon U.S. Eastern Time. TheraSphere should be administered using saline.

### 8.6 Residual measurement

Residual (i.e., non‐injected activity) within the activity vial and associated delivery apparatus must be measured and recorded. Currently, no consensus exists regarding the optimal method for residual quantitation. A common technique is to use exposure rate measurements at a known distance from the vial, contained within an acrylic jar large enough to hold the waste container, before and after administration. Residual measurements should be taken with the puncture‐proof jar with waste inside the acrylic container. Exposure rates are taken in four compass points or by slowly rotating the container at a fixed distance and an average exposure measurement is calculated. Each manufacturer provides a template for this measurement that may be used to ensure a reproducible setup.

## 9 POST‐TREATMENT IMAGING AND DOSIMETRY

### 9.1 Imaging considerations

Post‐treatment imaging is a valuable tool for treatment verification and absorbed dose calculation. Although pre‐treatment imaging with ^99m^Tc‐MAA may predict post‐treatment activity distributions within a patient, the actual distribution of ^90^Y microspheres can depend on numerous factors such as catheter placement, number of spheres, sphere specific gravity, changes in anatomy between mapping and treatment, and the variability of hemodynamics within a particular patient. Post‐treatment imaging provides an opportunity to observe and quantify the actual microsphere distributions. If a sub‐optimal microsphere distribution is obtained, post‐treatment imaging can guide subsequent interventions by ^90^Y microsphere re‐treatment or other therapies.

Imaging can be performed by SPECT/CT or PET/CT following administration of ^90^Y‐microspheres, with SPECT imaging often categorized as a primarily “qualitative” modality, whereas PET imaging has typically been described as inherently “quantitative”. Although the quantitative accuracy of ^90^Y PET/CT (via the rare internal pair production branch^64^), has been shown to provide superior accuracy in phantom studies,^65^, ^66^ both modalities have the potential to provide inherently quantitative ^90^Y image reconstructions.^67^, ^68^, ^69^, ^70^, ^71^ In terms of availability and propensity of use, SPECT/CT is more cost effective and generally more accessible in the community, and thus has been historically preferred over PET/CT for post‐radioembolization imaging. With that said, an increased focus on quantitative accuracy and dosimetry‐guided therapy may lead to increased adoption of PET/CT for post‐treatment imaging. Regardless of the modality used, post‐treatment imaging should be performed for the safety of the patient and purposes of treatment verification.

#### 9.1.1 SPECT/CT

Single photon imaging of ^90^Y is possible due to the generation of Bremsstrahlung radiation following beta decay. Due to the continuous nature of ^90^Y Bremsstrahlung radiation, typical scatter correction methods (e.g., the triple energy window [TEW] method) significantly under‐correct for scatter, and therefore the choice of scatter correction is the primary factor that contributes to the quantitative and spatial accuracy of ^90^Y SPECT images. Qualitative SPECT images should be acquired if ^90^Y‐specific scatter corrections are unavailable. However incomplete scatter correction results in reduced contrast between high‐ and low‐activity concentration structures. Medium energy (ME) or high‐energy (HE) collimators should be used in SPECT imaging. When choosing between the ME and HE collimators, clinicians may consider that although HE collimators result in reduced spatial resolution, the further reduction in septal penetration improves quantitative accuracy and contrast recovery. For either the ME or HE collimator, matrix sizes of ≥ 128 × 128 and total projections ≥ 120 provide optimal results. Several photon energy windows have been described in the literature. There is consensus that a ∼90‐160 keV window provides a reasonable balance between scatter fraction and sensitivity but no standard imaging protocol has been established.^68^, ^72^, ^73^, ^74^, ^75^ For qualitative purposes, images should be reconstructed according to institutional and physician preference. For quantitative analysis of post‐treatment imaging, a greater number of iterative updates (iterations multiplied by subsets) should be used with no additional filtering for noise reduction. The total number of subsets should be less than ∼0.25 times the total number of projections. Iterative updates in the range of ∼80–120 may provide a reasonable balance between spatial resolution and noise. For ^90^Y SPECT/CT acquisitions, a reconstruction algorithm can only be considered quantitative in nature if at least attenuation correction and an empiric scatter correction methodology are implemented.^68^ Facilities should perform their own independent phantom studies to verify energy window, collimator selection, and iteration number about relevant clinical endpoints. Such parameters should be kept constant for clinical use.

#### 9.1.2 PET/CT

Post‐treatment ^90^Y imaging with PET/CT is preferred over SPECT/CT, due to superior spatial resolution and reduced susceptibility to un‐corrected scatter. Although superior to SPECT imaging for ^90^Y quantitative imaging, the advantages of PET/CT are partially mitigated by the low internal pair production branching ratio (31.9 × 10^−6^ positrons per decay),^76^, ^77^ as well as a high single detection rate from Bremsstrahlung production. Due to these effects, the accuracy of dead time and random corrections are important. For this reason, it is advisable to evaluate the quantitative accuracy of a PET/CT system prior to performing patient studies. This can be accomplished by scanning a sealed vial of ^90^Y‐microspheres. For TheraSphere, the PMMA hand shield is adequate to induce annihilation of all positrons emitted. For SIR‐Spheres, it is recommended that the vial is placed within a flush‐fitting plastic container or submerged in water. Some degree of bias in activity quantification is expected^78^, ^79^ and correction for this effect should be made either by adjusting the isotope branching ratio within the scanner configuration (such that activity readings match the dose calibrator) or by applying a correction factor to images prior to use for dosimetry. Sites with multiple PET/CT scanners should be consistent in their method for correction of bias in quantitation. Sites administering both TheraSphere and SIR‐Spheres should independently characterize this effect for each sphere type.

PET/CT imaging with time‐of‐flight (TOF) enabled systems is preferable to non‐TOF enabled systems, as this has been shown to reduce noise and improve activity recovery.^80^ Filtration with 4–5 mm gaussian smoothing has been shown to improve agreement between measured and true calculated dose volume histograms (DVHs), although this is not appropriate when computing voxelwise absorbed dose by kernel convolution rather than local deposition.^42^, ^81^ Depending on scanner sensitivity and axial field‐of‐view, approximately 10–15 min per bed position with 3–4 bed positions is often adequate in terms of counting statistics and coverage of anatomy (liver or liver + lungs).

### 9.2 Dosimetry

Although pre‐administration treatment planning and dosimetry may sometimes correspond well with the true therapeutic activity and absorbed dose distribution, numerous factors can result in differences between the planned and delivered absorbed dose distribution. Several of these factors include physical differences between ^99m^Tc‐MAA and microspheres (specific gravity, sphere number, particle size distribution), differences in infusion location within the hepatic arterial supply, biological differences during the infusion (vessel behavior, anatomic changes between mapping and therapy), as well as the simple statistical distribution probability of individual microspheres. For these reasons, post‐treatment dosimetry can provide actionable information regarding compliance with the initial treatment plan, allowing for evaluation of target coverage, adequate target dosimetry, as well as potential off‐target exposure. If post‐treatment dosimetry for purposes of routine patient management is calculated, it must be performed using quantitative SPECT or PET images. Planar gamma scintigraphy or SPECT/CT without advanced scatter correction is not considered quantitative. Post‐treatment absorbed dose calculations may be performed following any of the established dosimetry techniques described in section 5 of this document. For PET‐based post‐treatment dosimetry, conditions for relative dosimetry may be met. However, dosimetry may also be performed on an absolute activity measurement basis, provided that the QMP has evaluated the system for quantitative bias, as described in section 7.A above. Methodology type and reconstruction parameters should be documented. Some centers without these capabilities may require dosimetric information in certain situations such as suspected reportable medical events. In such cases, low accuracy dosimetry information obtained from non‐quantitative ^90^Y Bremsstrahlung imaging may be reported but should be properly documented as a qualitative estimate.

### 9.3 Compliance with the written directive

Compliance with the AU's WD must be evaluated in relation to criteria for reportable medical events within the institution's NRC or Agreement State licensing jurisdiction. This evaluation must consider all aspects of the procedure (correct quantity of radioactivity, correct administration route, correct patient, and correct isotope and microsphere product) as described in Section 2. Post‐treatment imaging plays a leading role for evaluation of whether the intended tissues received microspheres, and whether off‐target tissues received significant quantities of radioactivity. Although post‐treatment dosimetry is not required per FDA labeling for either ^90^Y microsphere product, institutions must be equipped to estimate absorbed dose (or administered activity if using the BSA model) if conditions for classification as a reportable medical event are met. This standard will typically require some type of post‐treatment imaging for all patients to ensure delivery to the appropriate site.

## 10 PATIENT SAFETY

### 10.1 Patient release criteria

Pursuant to 10.CFR.35.75,^82^ a patient treated with ^90^Y can be released if the total effective dose equivalent to another person does not exceed 5 mSv.

Zanzonico et al^83^ estimated that an activity of 1420 GBq (38.4 Ci) is required to reach this regulatory limit by considering only the Bremsstrahlung radiation and assuming an occupancy factor of 0.25 at 1 m distance. This is a conservative limit as the attenuation of photons in the body was not considered and a point‐like source model was assumed in this calculation. It is very unlikely that this limit is reached since administered activities usually do not exceed 20 GBq (540 mCi).

Gulec et al^84^ calculated the effective dose to others as 0.011 mSv, using the same occupancy and distance, due to Bremsstrahlung from a patient treated with a clinically relevant 3 GBq activity of ^90^Y. When the distance is decreased to 0.3 m (a travelling or sleeping partner), the effective dose to the partner was calculated as 0.11 mSv. The effective dose to an infant at a distance of 0.1 meter with an occupancy factor of 1 hour per day was calculated as 0.05 mSv. In the case of a nursing infant, effective doses of 0.02 and 0.18 mSv were estimated by Gulec due to the ingestion of breast milk and external exposure while feeding, respectively.

### 10.2 Patient instructions

Patient instructions are required only if the effective dose to other individuals is likely to exceed 1 mSv (0.1 rem). This effective dose would be one fifth of the maximum allowable release value, corresponding to an administered activity of 284 GBq (∼7.7 Ci) per Zanzonico et al,^83^ far in excess of normal clinically relevant activities. While not required, release instructions should be provided out of an abundance of caution and as educational material. Instructions should address relevant potential risks such as sleeping arrangements, post‐mortem or surgical resection information, travel restrictions, and special considerations for children and pregnant women.

**FIGURE 2:**
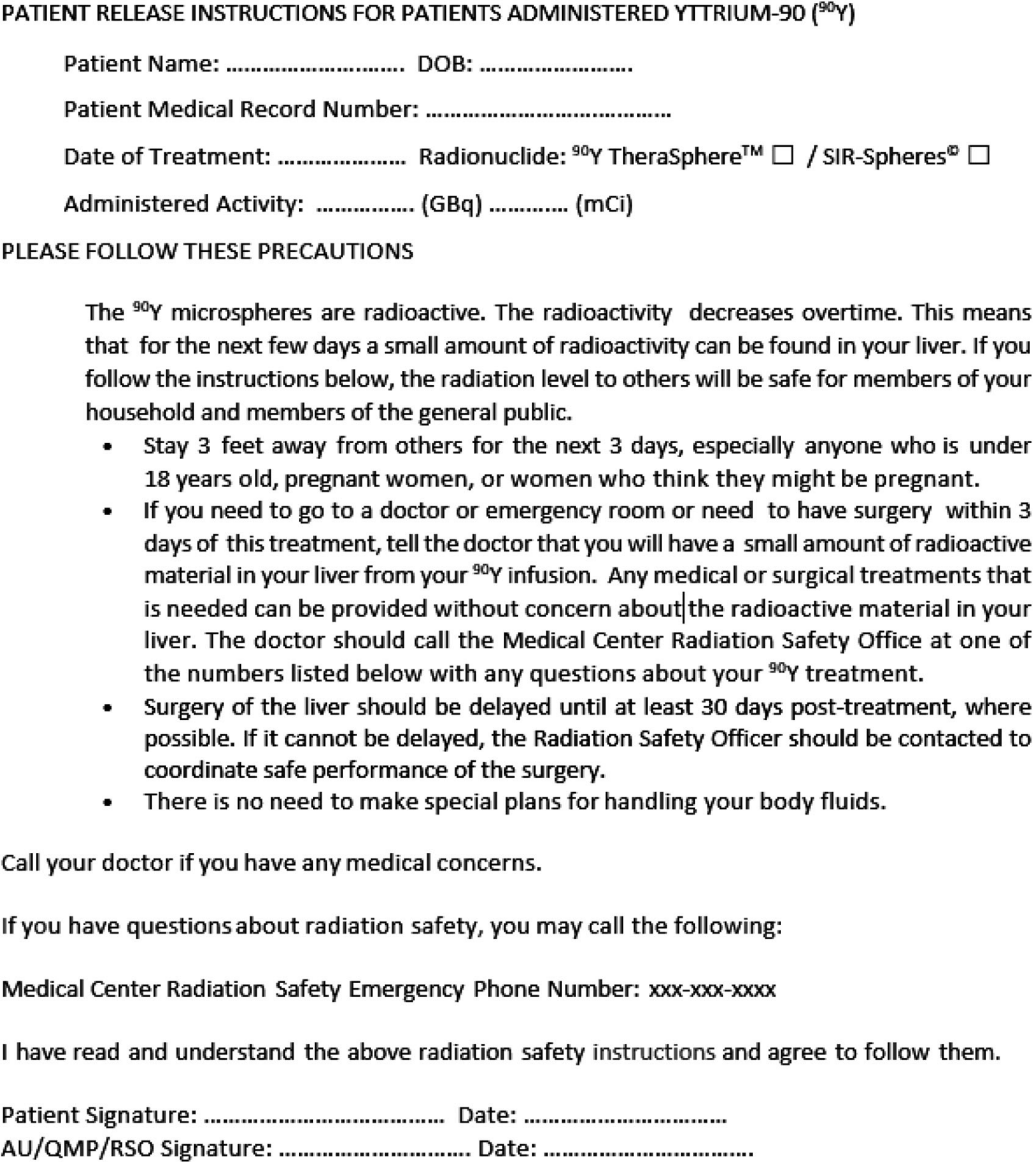
An example of patient release instructions containing the necessary information is shown.

**FIGURE 3:**
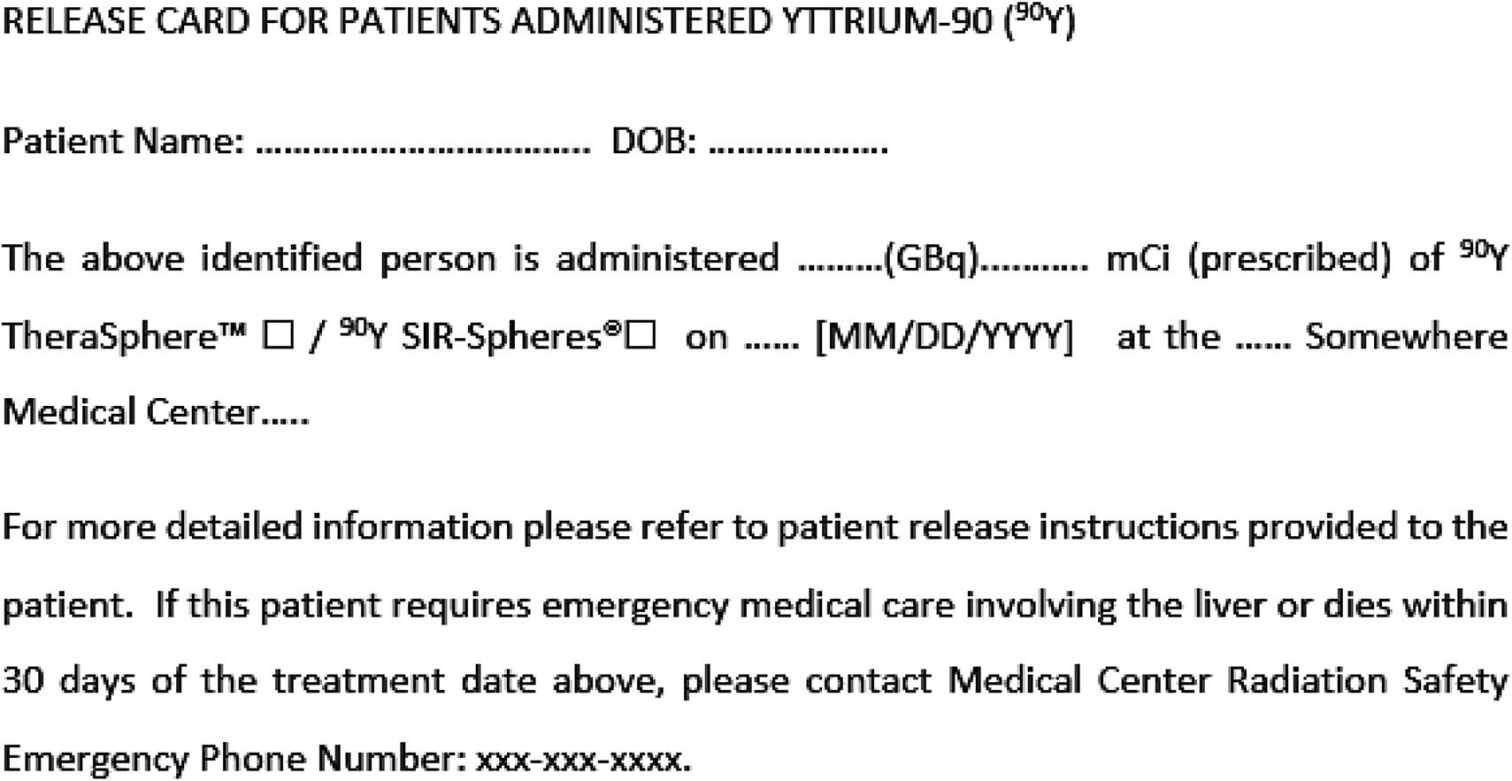
Example of patient release card.

Per 10 CFR 35.75 (c),^78^ a record of the basis for authorizing the release of an individual in accordance with section 10.CFR.35.2075(a)^85^ must be maintained. A template of release information sheets can be found in USNRC Regulatory Guide 8.39^86^ for adaptation and is included in Figure 2 below. The patient should be provided with a card indicating that they underwent a ^90^Y hepatic radioembolization procedure. A sample card is shown in Figure 3.

Patients and their caregivers should be reminded to refer to the release instructions and card if the patient experiences a medical emergency. Any treatment other than those involving the liver may be provided without concern about the radioactivity.

Patients treated with ^90^Y radioembolization may become candidates for surgery. It is recommended to delay surgery for 10 half‐lives (640 h, ∼27 days) after ^90^Y administration for radiation safety considerations. The decision must be made collaboratively with the care team to ensure quality and safety are not compromised. If the interval between the ^90^Y treatment and the surgery is less than 27 days, a QMP, CHP, RSO, or other radiation staff member must attend the surgery, make an informed determination of the level of remaining activity, advise the surgical team of his/her findings and any recommended precautions. If liver tissue is removed during the surgery, the QMP, CHP, RSO, or other radiation staff member must survey it and follow appropriate radioactive waste disposal procedures as necessary.

## 11 POTENTIAL FAILURE MODES

A recent survey by Smith et al.^87^ showed 152 medical events involving ^90^Y between 2008 and 2017. 85.8% of these medical events resulted in deviation from the prescription while 12.4% resulted in treatment of the wrong site. In the remaining 1.8%, the wrong patient was treated. The authors reported that 51% of the medical events were due to human error, 38% were due to equipment failure, 25% were due to procedure problems, and 17% were due to patient action (not intervention). The remaining percentage of the causes were classified as other or not reported at all.

Recognizing potential failure modes and understanding their sources is crucial for building a safe and effective program. A failure modes and effects analysis (FMEA) can help with identifying potentially high‐risk failure modes. Younge et al. performed an FMEA for their dual‐product ^90^Y microsphere program.^88^ Table 4 is a summary of the results from their study. While these results can be helpful with building or improving programs, they are unique to that institution, and all institutions should perform an FMEA of their own ^90^Y microsphere programs to improve their robustness and safety. A key take‐away from all high‐risk failure modes listed below is they could be mitigated with a combination of:

1. *Training of personnel involved in ^90^Y microsphere treatments*

**TABLE 4:** Summary of FMEA performed by Younge et al.^88^ for each step of ^90^Y microsphere therapy.

Failure mode	Cause	Clinical impact
*1. Pre‐treatment imaging*		
scheduling the incorrect microsphere type	typographic error inadequate experience/training of the AU and/or IR	major delay of patient treatment
insufficient information on the planning study		
incorrect measurement/recording of lung shunt fraction (LSF)		lung toxicity or incorrect identification of patient as ineligible
incorrect target identified		under‐dosing or missing the target
*2. Treatment planning*		
dosimetry worksheet error	inadequate experience/training typographic errors	patient toxicity tumor under‐/over‐dosing
treatment volume measured/recorded incorrectly		
incorrect dose range used for cirrhotic/non‐cirrhotic patient		
incorrect LSF entered in dosimetry worksheet		
previous treatment not considered		patient toxicity
gastrointestinal shunt not considered/recorded incorrectly		
planning performed for wrong type of microspheres		patient toxicity tumor under‐/over‐dosing major delay to the patient's treatment
*3. Dosage Ordering*		
dosage not ordered or ordered late	delays in planning communication error	major delays to the patient's treatment
incorrect delivery date/time or incorrect requested calibration date/time	inadequate experience/training typographic errors	
incorrect order form submitted		
incorrect type of microspheres ordered		
*4. Dosage Preparation*		
incorrect assay date/time recorded on check‐in paperwork	inadequate experience/training typographic errors	patient toxicity tumor under‐/overdosing
incorrect infusion date/time recorded on check‐in paperwork		
incorrect dose calibrator factor used for assay		
incorrect patient dosimetry worksheet used		
not using aseptic techniques when assaying/preparing dosage		
*5. Treatment administration*		
wrong patient	inadequate experience/training typographic errors communication error	patient toxicity tumor under‐/overdosing
catheter incorrectly placed (i.e., wrong site)		
incorrect vial/dose used		
kinks/resistance/clogs in administration catheter after treatment initiation	inadequate experience/training	
vendor‐specific instructions not followed for administration		
lung shunting not verified pretreatment	inadequate experience/training communication error	patient toxicity
system disconnection (lines disconnecting, needles pulled out of vial, etc) during or after administration		patient toxicity tumor under‐/overdosing radioactive contamination of area (additional exposure to staff, patient and delay for cleanup)

Vendor‐provided training is a resource upon starting a ^90^Y program. Ongoing training may also be available on an as‐needed basis either by experienced in‐house staff (e.g., QMP, CHP, RSO) or vendor. It is imperative that all individuals involved in the treatment administration (AU, Interventional Radiologist, QMP, CHP, RSO, other radiation staff member, technologists, etc.) receive this training and are familiar with the technical steps, potential issues, and trouble‐shooting that the procedure involves. A credentialing form can be useful for tracking training of individuals on the team and documenting that all aspects of the treatment have been discussed and understood by the team, including mitigation of potential sources of errors.

2 *Built‐in second checks, checklists, and time‐outs*

A written standard of practice procedure should include a detailed outline of the clinical workflow, from scheduling to RAM waste handling, with each team member's responsibilities clearly outlined. Within this workflow, second checks of other team members, especially when working across disciplines, can help avoid typographic errors or lapses in judgment. This could include AU and Interventional Radiologist verifying the treatment site and prescription with each other; QMP, CHP, RSO, or other radiation staff member verifying the treatment plan created by a dosimetrist or another QMP; and so forth. Step‐by‐step checklists before, during, and after the procedure help minimize potential sources of errors. It is especially useful to have an individual read the checklist aloud in the procedure room for all individuals to hear and follow prior to start of ^90^Y administration.

3 *Communication amongst team members*

Given its inter‐disciplinary nature, facile communication needs to be established and respected from patient scheduling through room survey, decontamination, and patient release. This should be defined and outlined in the written standard of practice procedure. An electronic white‐board system can help facilitate, track, and document this communication throughout the clinical workflow. Team members should feel comfortable speaking up if they notice an error at any point during the procedure.

4 *Monitoring vendor warnings and national databases for potential errors and failure modes*

Error reporting by individual institutions, as recorded in national databases and vendor websites, can greatly reduce the chance of similar events at other institutions. Programs should monitor these databases and evaluate/adapt their processes as needed to improve patient and staff safety.

## 12 SUMMARY

Yttrium‐90 radioembolization is a real time image‐guided brachytherapy procedure used for the treatment of liver tumors that takes advantage of the dual hepatic blood supply. Yttrium‐90 microspheres suspended in a colloid solution are injected into the tumor site under image guidance. This report provides detailed support to institutions intending to establish such a program and those who already have a program and wish to develop it further. An introduction is provided that deals with preparatory steps, patient selection, and a procedure flowchart. Information is provided to hospital administrators regarding the skill sets required for safe, efficacious, and correct implementation of such a complex clinical procedure, along with economic aspects associated with a ^90^Y program.

Regulatory compliance, staff training, and technologies needed for ^90^Y are addressed in detail. Pre‐procedure imaging to determine tumor burden, lung shunt to limit pulmonary toxicity, planned vascular approach to the tumor site, and required ^90^Y activity to deliver a tumoricidal absorbed dose are explained in separate sections. Post‐treatment imaging and dosimetry are included as important quality assurance measures to quantify attainment of treatment goals. Patient and staff safety and associated precautionary radiation safety measures are addressed in detail.

Cognizant of the complexity of such a procedure, special attention is given to the potential of failure modes. These are addressed in Section 9, along with causes that may give rise to them. Advice is provided regarding their clinical impact, with measures to prevent and ameliorate their consequences.

It is hoped that this report is of value to the wide spectrum of professionals (medical physicists, interventional radiologists, radiation oncologists, RSOs, regulatory staff, etc.) involved in ^90^Y treatment planning and delivery. Current medical physics support for ^90^Y is widely variable. This report aims to set a minimum practice standard for individuals, institutions, and regulatory bodies.

This working group has benefited from the support of the AAPM Subcommittee on Practice Guidelines and the AAPM at large. Members of the Society of Interventional Radiology provided helpful feedback. To all, we extend our gratitude.

## AUTHOR CONTRIBUTIONS

This guideline was reviewed and updated by Medical Physics Practice Guideline Task Group 356 of the Professional Council of the AAPM. Each author reviewed recent literature on the topic and offered opinions on language for the guideline. All authors contributed to the writing of the document. They also reviewed and applied comments from the full AAPM membership to the document.

This guideline was developed by the Medical Physics Practice Guideline Task Group 356 of the Professional Council of the AAPM.

TG356 MPPG 14.a Members:

Nathan C. Busse, MS, Co‐Chair

Muthana S.A. L. Al‐Ghazi, PhD, FCCPM, FAAPM, FIOMP, Co‐Chair

Nadine Abi‐Jaoudeh, MD, FSIR, CCRP

Diane Alvarez, MS

Ahmet S. Ayan, PhD

Erli Chen, MS, FAAPM, FACR

Michael D. Chuong, MD, FACRO

William A. Dezarn, PhD

Shirin A. Enger, PhD

Stephen A. Graves, PhD

Robert F. Hobbs, PhD

Mary Ellen Jafari, MS, FACR, FAAPM

S. Peter Kim, MEng

Nichole M. Maughan, PhD

Andrew M. Polemi, PhD

Jennifer R. Stickel, PhD

**AAPM Subcommittee on Practice Guidelines**—AAPM Committee responsible for sponsoring the draft through the process:

Daniel C. Pavord, MS, FAAPM, Chair

Mary Ann Keenan, DMP, Vice‐Chair—Imaging

Arthur J. Olch, PhD, FAAPM, Vice‐Chair—Therapy

Muthana S.A.L. Al‐Ghazi, PhD, FCCPM, FAAPM, FIOMP

Nathan C. Busse, MS

Leigh A. Conroy, PhD

Ryan F. Fisher, PhD

Jonas D. Fontenot, PhD, FAAPM

Mark W. Geurts, MS

Eric L. Gingold, PhD, FAAPM

Per H. Halvorsen, MS, FAAPM, FACR

Siyong Kim, PhD, FAAPM

Robert F. Krauss, DMP

Susan L. Richardson, PhD, FAAPM

John M. Wait, MS

Nicholai Wingreen, AAPM Staff

## CONFLICT OF INTEREST STATEMENT

Members of TG356 listed below attest that they have no potential Conflicts of Interest related to the subject matter or materials presented in this document: Muthana Al‐Ghazi, Ahmet Ayan, Nathan Busse, Erli Chen, Shirin Enger, Stephen Graves, Robert Hobbs, Mary Ellen Jafari, Peter Kim, Nichole Maughan, Andrew Polemi, Jennifer Stickel.

Members of TG356 listed below disclose the following potential Conflict(s) of Interest related to subject matter or materials presented in this document: Nadine Abi‐Jaoudeh and Diane Alvarez received research funding from Sirtex Medical. Michael Chuong served as a speaker for Sirtex Medical. William A. Dezarn served as a consultant to Sirtex Medical.

## DISCLOSURE STATEMENT

The Co‐Chairs of Medical Physics Practice Guideline 14 Yttrium‐90 Radioembolization (TG356) have reviewed the required Conflict of Interest statement on file for each member of TG356 and determined that disclosure of potential Conflicts of Interest is an adequate management plan.

## Footnotes

a The MIRD method in the context of this document refers to TARE dosimetry methods that assume local deposition of energy. Although the MIRD committee has published a S‐value convolution methodology, this methodology is considered a dose‐kernel‐based simulation rather than a MIRD method in the context of this document.^40^
